# Should oral anticoagulant therapy be continued during dental extraction? A meta-analysis

**DOI:** 10.1186/s12903-016-0278-9

**Published:** 2016-08-26

**Authors:** Shuo Yang, Quan Shi, Jinglong Liu, Jinru Li, Juan Xu

**Affiliations:** 1 Department of Stomatology, Chinese People’s Liberation Army General Hospital, 28 Fuxing Road, Beijing, 100853 China; 2 Department of Stomatology, Chinese People’s Liberation Army 322 Hospital, 2 Yunzhong Road, Datong, 037000 China

**Keywords:** Oral anticoagulant therapy, Dental extraction, Bleeding, Meta-analysis

## Abstract

**Background:**

Oral anticoagulation therapy is widely used to reduce the risks of thromboembolism. However, the therapy increases the risk of hemorrhage during the surgical procedures. The aim of this meta-analysis was to evaluate the bleeding risk of patients continuing or discontinuing oral anticoagulant therapy while undergoing dental extractions.

**Methods:**

Six electronic databases, including PubMed, Embase, Cochrane library, Web of Science, China Biology Medicine disc (CBM), and China National Knowledge Infrastructure (CNKI), were searched in March, 2016. Relevant articles were screened by two independent reviewers under our inclusion criteria. Quality was evaluated using the Cochrane Collaboration risk of bias tool. Meta-analyses were conducted with fixed and random effects models as appropriate.

**Results:**

Six studies (with a total of 591 patients) were included in our meta-analysis. Our results showed that there was no significant difference in the bleeding risk between patients continuing or discontinuing oral anticoagulant therapy while undergoing dental extractions (risk ratio, 1.31; 95 % CI, 0.79, 2.14; *P* > 0.05). There was also no significant difference in bleeding risk 1 day (risk ratio, 0.91; 95 % CI, 0.35, 2.37; *P* > 0.05) and 7 days (risk ratio, 1.47; 95 % CI, 0.83, 2.59; *P* > 0.05) after the dental extraction.

**Conclusion:**

Under current studies and evidence, it appears that patients continuing oral anticoagulant therapy do not have an increased risk of bleeding after dental extractions compared to patients who discontinue oral anticoagulant therapy.

## Background

With the living standard gradually increasing and diet habits continuously changing, cardiovascular diseases, such as mechanical heart valve, atrial fibrillation and venous thromboembolism, have become more and more common. Millions of people receive oral anticoagulant therapy (OAT). Used to reduce thrombosis, oral anticoagulation therapy is one of the most effective prophylactic medications for preventing life-threatening events [1, 2]. Oral anticoagulant drugs mainly include heparin, warfarin and new oral anticoagulants (NOACs) [3]. Heparin should be administered intravenously, and its action is to interfere with thrombin-antithrombin pathways and reduce fibrin formation. Warfarin, which is derived from 4-hydroxycoumarin, is a competitive inhibitor of vitamin K. It is widely used all over the world [4]. New oral anticoagulants (NOACs) include factor Xa inhibitors, such as rivaroxaban, apixaban, and edoxaban, which mainly inhibit the factor Xa activity of the prothrombinase complex in the propagation phase, and factor IIa inhibitors such as dabigatran. Although their advantages included a rapid onset and a short half-life, NOACs are still not ready to completely replace conventional anticoagulants [3].

However, with the wide use of oral anticoagulant therapy (OAT), a major disadvantage of OAT that should receive more attention, is the increased risk of hemorrhage during surgical procedures. OATs are frequently prescribed to elder patients, who also have a higher demand for dental extraction caused by caries, periodontitis or other dental diseases. So, the problem of postoperative bleeding becomes a major concern for the dentist. Whether to continue or discontinue OAT has become a strong controversy. If OAT is continued, there is a high risk of bleeding, and if OAT is discontinued, the thromboembolic complications are potentially deadly.

Discontinuing OAT 2 or 3 days before oral surgery has been a widely used strategy for managing patients on warfarin [5, 6]. However,the risk of postoperative bleeding apparently does not decrease, and the thromboembolic risk increases [7]. There have been documented incidences of thromboembolic events when warfarin was stopped prior to a dental procedure or a minor oral surgery [8, 9]. Garcia et al. [10] published the first important prospective study on the risk of thromboembolism with a short-term interruption of OAT by reporting that the incidence of thromboembolism within a 30-day follow-up period was 0.5 %. Though the incidence of thromboembolism is low, the consequence may be deadly. These thromboembolic events can have devastating clinical consequences, such as an embolic stroke, which can result in major disability or death, or myocardial ischaemia, which can increase risk of death two to four fold [11]. To solve this problem, some strategies have been developed in the last few decades. These strategies include reducing the dose of the anticoagulant drugs [12, 13] or bridging it with heparin [14, 15]. These two methods, however, do not completely eliminate the risk of thromboembolic events, such as stroke [9]. Currently, there are a lot of publications suggest that dental extractions may be carried out with no OAT interruption if local hemostasis is adequately maintained in the OAT patients [14, 16–20]. Nevertheless, there is still a lack of public recognition of the treatment guidelines for treating these patients. To address this controversy and to provide evidence-based recommendations, we have completed a meta-analysis. Articles on randomized controlled trials (RCTs) or controlled clinical trials (CCTs), including patients under OAT who undergo dental extractions were collected, and the postoperative bleeding was compared between patients who continued OAT and patients who discontinued OAT.

## Methods

The methods for this review were based on the Cochrane Handbook for Systematic Reviews of Interventions. Throughout the whole process, the studies were assessed by 2 observers independently, and any disagreement was resolved by discussion.

### Database search

A comprehensive search of the PubMed, EMBASE, Web of Science, Cochrane Library, China Biology Medicine disc (CBM), and China National Knowledge Infrastructure(CNKI) databases was conducted in March 2016. The search strategies for PubMed, EMBASE, Web of Science and Cochrane Library are shown in Table 1.

**Table 1:** The key words, database and search result

Database	Key words	Result
Pubmed	(oral anticoagulant therapy OR OAT OR anticoagulant*) AND (dental surgery OR dental extraction* OR tooth extraction*)	879
Embase	(oral anticoagulant therapy OR OAT OR anticoagulant*) AND (dental surgery OR dental extraction* OR tooth extraction*)	634
Cochrane Library	(oral anticoagulant therapy OR OAT OR anticoagulant*) AND (dental surgery OR dental extraction* OR tooth extraction*)	2
Web of Science	(dental surgery OR dental extraction* OR tooth extraction*) AND (oral anticoagulant therapy OR OAT OR anticoagulant*)	664
Total		2179

### Study selection

Two reviewers independently evaluated all of the search results, and the inclusion criteria were as follows:

1. Study design—studies were designed as randomized controlled trials (RCTs) or controlled clinical trials (CCTs)
2. Participants—patients were receiving OAT and required dental extractions
3. Comparators—the postoperative bleeding between patients who continue or discontinue OAT.
4. Outcomes—postoperative bleeding (spontaneous bleeding, induced bleeding and minor bleeding)

The exclusion criteria were as follows:

1. In vitro study (laboratory studies or animal studies), case reports or letters.
2. Studies on antiplatelet medications.
3. Study outcomes were not clearly reported or the data could not be used for our meta-analysis.

### Data extraction

The following parameters were extracted from each of the selected studies: the first author, country, year of publication, design type, patient characteristics, intervention method, and bleeding outcomes. The information is recorded in the table.

Because there is no accepted standardized definition of bleeding outcomes for patients undergoing surgical procedures, we aimed to use the postoperative bleeding to summarize the bleeding outcome after thoroughly reading the included articles. The bleeding outcomes can be defined as two processes during the follow-up days. One is the patient’s own perceived bleeding, such as spontaneous bleeding that continues for more than 20 min [21] and oozing from the extraction site [7]. The other bleeding outcome is examined by doctors during the appointment, and the presence of a solid clot covering the extraction socket was considered as no bleeding, while the presence of a fresh clot that shed easily or oozing blood was considered to be positive bleeding [4].

### Risk of bias evaluation

The following seven items were taken into consideration:

(1) allocation concealment, (2) random sequence generation, (3) blinding of participants and personnel, (4) blinding of outcome assessment, (5) incomplete outcome data, (6) selective reporting, and (7) other bias. The risk of bias for each item was judged as low risk, high risk, or unclear risk. The overall risk of bias for each study was evaluated by the following criteria: If the risk of bias was low for all the items, the study was of low risk. If one (or more than one) of the risk factors for bias was high for key items, the study was of high risk. If one (or more than one) of the risk factors for bias was unclear, the study had an unclear risk.

### Statistical analysis

The bleeding outcomes of the included studies were combined, and a meta-analysis was performed using RevMan software (version 5.3). Subgroup analyses were performed once the included studies had the same evaluation intervals. The risk of bleeding for continuing or discontinuing OAT was expressed as a relative risk (RR) with an associated 95 % CI. In addition, a chi-square and I^2^ test were used to estimate the degree of heterogeneity, with values of 25, 50, and 75 % corresponding to the low, moderate, and high heterogeneity, respectively. Substantial heterogeneity was defined as a P value <0.05 and I^2^ >50 %. The fixed-effects model was applied when I^2^ <50 %, and the random-effects model was applied when I^2^ >50 %.

## Results

### Literature search

**Fig. 1:**
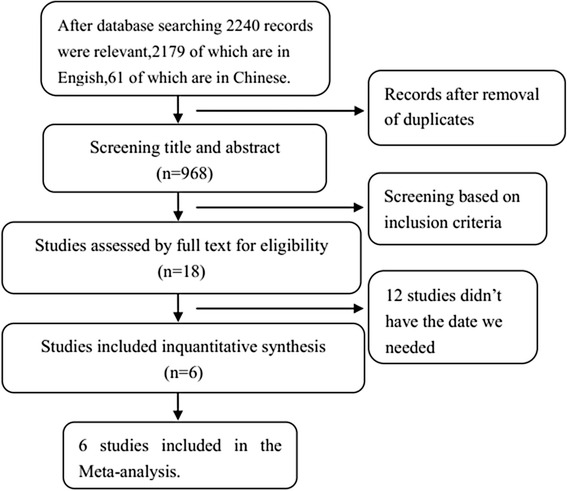
Study flow diagram

The search process is shown in Fig. 1. There were 968 relevant studies identified during the database search, and after the exclusion of duplication as well as titles, abstracts, and full-text screening, finally, we included six studies [4, 7, 16, 21–23] in our meta-analysis. Five of the studies are in English, and the other one [22] is in Chinese.

### Data extraction

The characteristics of the selected six studies are summarized in Tables 2 and 3. The studies were published between 1993 and 2010. Four of the six studies were RCT, and the other two were CCT. The total number of patients under OAT who received dental extractions was 581. The patients were divided into the following two groups: an OAT continued group and an OAT discontinued group. In the OAT continued group, patients did not stop their anticoagulant therapy when they needed dental extractions. In the OAT discontinued group, patients stopped their anticoagulant therapy 2 or 3 days before the dental extraction procedure and usually resumed the therapy on the same day. Four of the studies used warfarin as the anticoagulant drug, one used a vitamin K inhibitor and one had not report the anticoagulant. The details of the bleeding outcomes are shown in Table 3. The follow-up ranged from 1 to 7 days. The bleeding outcomes during patients’ review visits were recorded if the article mentioned.

**Table 2:** Characteristics of included studies

Study	Country	No.(T/C)^a^	Design	Anticoagulants	Average age (mean ± SD or mean (range))
Borea (1993) [21]	Italy	30(15/15)	RCT	NR^b^	T: 62.7 ± 6.1 C: 61.1 ± 10
Gaspar (1997) [23]	Israel	47(32/15)	CCT	Vitamin K inhibitor	T: 61.1(34–85) C: 53.4(35–72)
Devani (1998) [7]	UK	65(33/32)	CCT	Warfarin	T: 62.3(30–82) C: 61.3(32–81)
Evans (2002) [16]	UK	109(57/52)	RCT	Warfarin	T: 67(36–92) C: 66 (30–93)
Al-Mubarak (2007) [4]	Saudi Arabia	214(110/114)	RCT	Warfarin	T: 51.7 ± 14.7 53.1 ± 13.7
					C: 52.3 ± 14.3 48.7 ± 13.1
Duan XQ (2010) [22]	China	116(67/49)	RCT	warfarin	T: 59.5 ± 12.6 C: 61.5 ± 11.3

^a^ *T*treatment group, continue oral anticoagulant therapy group, *C* control group, discontinue oral anticoagulant therapy group

^b^ *NR*not report

**Table 3:** Characteristics of included studies

Study	Group	INR (mean/range)	Intervention method	Number of extraction	Follow up	Bleeding outcomes
Borea (1993) [21]	T: continued OAT. C: discontinued OAT.	T: 3–4.5 C: 1.5–2.5	T: Sutures and TA irrigation at surgery and MS for 7 day. C: Sutures and physiologic irrigation at surgery and MS for 7 days.	Single dental	7 days	Day 1: Spontaneous bleedings T: 1/15 C:2/15 Induced bleedings T:1/15 C:0/15 Day 2 to day 7: none
Evans (2002) [16]	T: continued OAT. C: discontinued OAT 2 days before extractions and resumed on the same day.	T: 2.5(1.2–4.7) C: 1.6(1.2–2.3)	All groups: Oxycellulose dressing and sutures for all patients.	T: 2(1–7) C: 3(1–9)	7 days	Postoperative bleeding T:15/57 C:7/52
Devani (1998) [7]	T: continued OAT. C: discontinued OAT 2 days before extractions.	T: 2.7(2.2–3.9) C: 1.6(1.2–2.1)	All groups: Placing haemostatic pack and sutures.	T: 2.1(1–9) C: 2(1–9)	5 days	Minor bleeding: Day 1 T:0/33 C:0/32 Day 2 T:0/33 C:1/32 Day 3 T:1/33 C:0/32 Day 4 to day 5:none
Gaspar (1997) [23]	T: continued OAT C: discontinued OAT 3 days before and resumed the same day	T: 2.5(1.9–3.5) C: 1.4(1.3–1.9)	All groups: Sutures and TA irrigation at surgery and MS for 2 min.MS for 7 days postoperativedays	NR	7 days	Postoperative bleeding T:2/32 C:1/15
Al-Mubarak (2007) [4]	T: continued OAT (group2 no sutures,group4 sutures). C: discontinued OAT 2 days before and resumed 12 h after dental extractions (group1 no sutures, group 3 sutures).	T: 1.85(1.4–2.3) C:2.55(1.9–3.1)	Local pressure (all) and sutures (group 3 and 4).	All patients range 1–5, single extraction (63.3) two teeth (25 %), three teeth (7.5 %) four teeth (3.3 %) and five teeth (0.8 %).	7 days	Postoperative bleeding T:8/110 C:7/114
Duan XQ (2010) [22]	T: continued OAT. C: discontinued OAT 3 days before and resumed the next day.	ALL: 1.80–2.67	All groups: Local pressure and sutures.	T:1.5(1–5) C:1.3(1–5)	1 day	Postoperative bleeding T:6/67 C:5/49

*OAT* oral anticoagulant therapy, *T* treatment group, *C* control group, *INR* international normalized ratio, *MW* mouthwash, *TA* tranexamic acid, *NR* not report

### Risk of bias evaluation

**Fig. 2:**
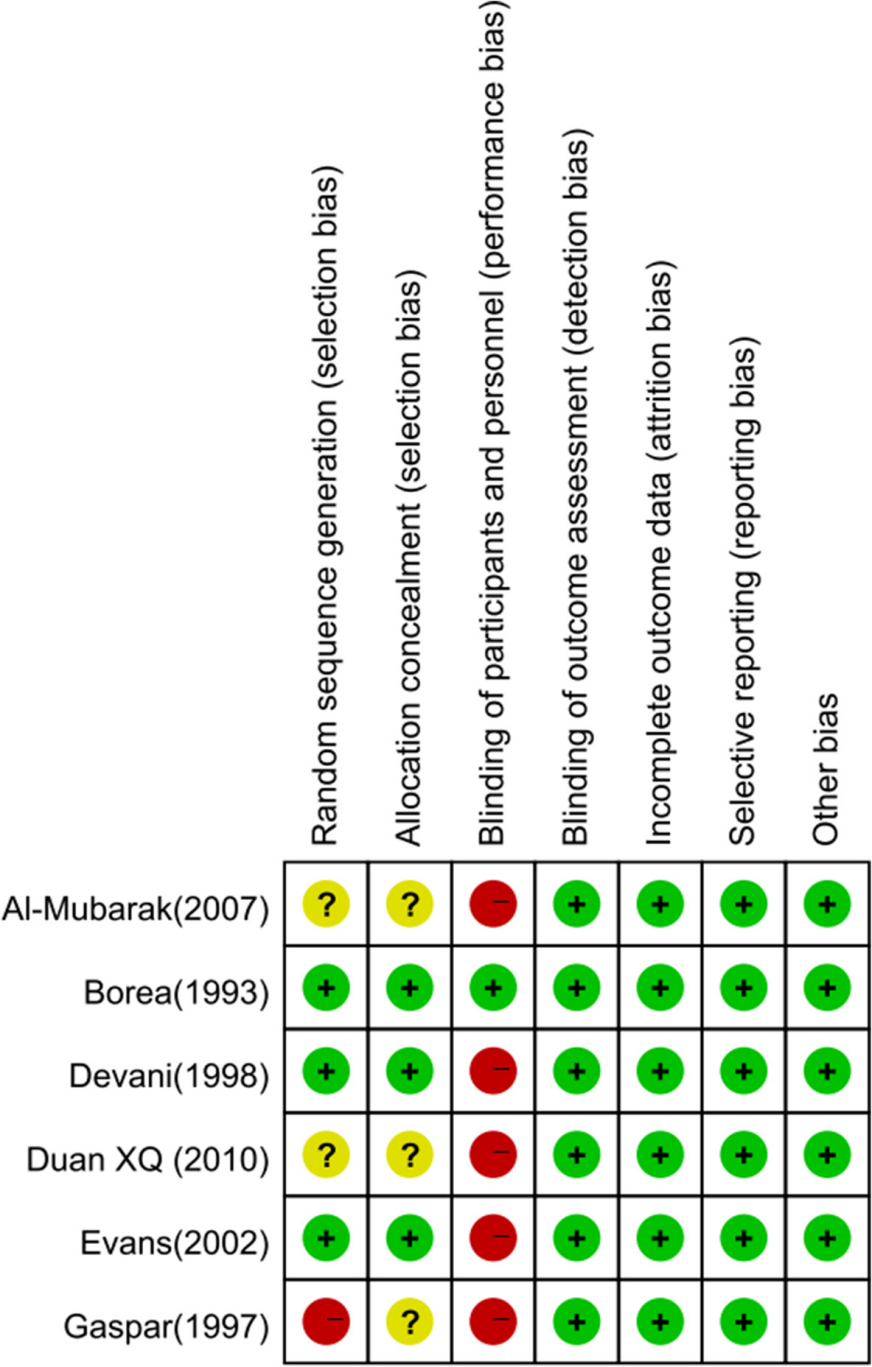
Risk of bias of the studies. Summary of risk of bias for included studies. Green indicates a low risk of bias, yellow indicates an unclear risk of bias, and red indicates a high risk of bias

The risk of bias summary is shown in Fig. 2. Of the six included studies, one [21] was judged to have a low risk of bias because all of the items had a low risk of bias. Five [4, 7, 16, 22, 23] of the studies were judged to have a high risk of bias because all of the studies failed to blind participants and personnel, and three [4, 22, 23] studies failed to describe the method of randomization and had no report of the allocation concealment.

### Meta-analysis

According to the postoperative bleeding outcomes, we performed a meta-analysis to compare the bleeding risk of the OAT continued and OAT discontinued groups. Six studies were included, with 314 subjects in the treated group and 277 in the control group.

**Fig. 3:**
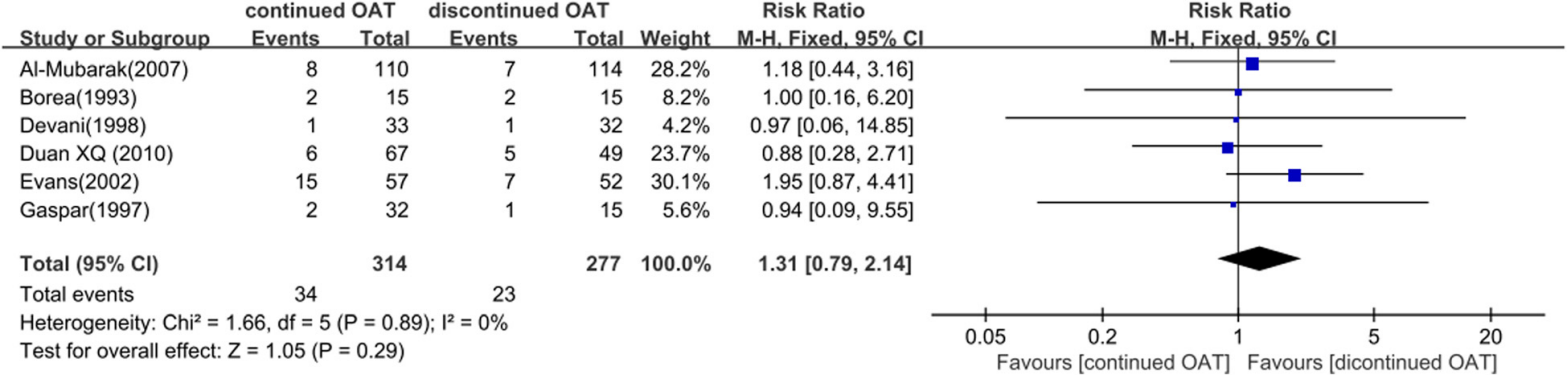
Forest plot of the difference of postoperative bleeding between OAT continued or discontinued group

The incidence of postoperative bleeding was 10.8 % (34/314) in the OAT continued group and 8.30 % (23/277) in the OAT discontinued group. A fixed-effects model was applied because of the low heterogeneity across the studies. There was no significant difference in bleeding risk between the OAT continued group and OAT discontinued group (*P* = 0.29). The risk ratio was 1.31 and 95 % CI (0.79, 2.14) (Fig. 3).

**Fig. 4:**
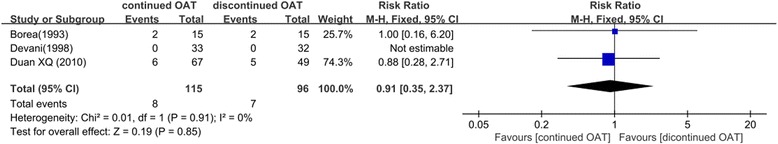
Forest plot of the difference of postoperative bleeding between OAT continued or discontinued group 1 day after the surgery

Subgroup analyses were performed when three or more studies recorded the bleeding incidence using the same evaluation intervals. Two time points (1 and 7 days after the operation) fit the criteria. Three studies [7, 21, 22], with 115 subjects in the treated group and 96 in the control group were included in the 1 day subgroup. The bleeding occurred in 8 of 115 (6.9 %) patients in the treated group and in 7 of 96 (7.2 %) in the control group. A fixed-effects model was applied, and there was no significant difference between the two groups (*P* = 0.85). The risk ratio was 0.91 and 95 % CI (0.35, 2.37) (Fig. 4).

**Fig. 5:**
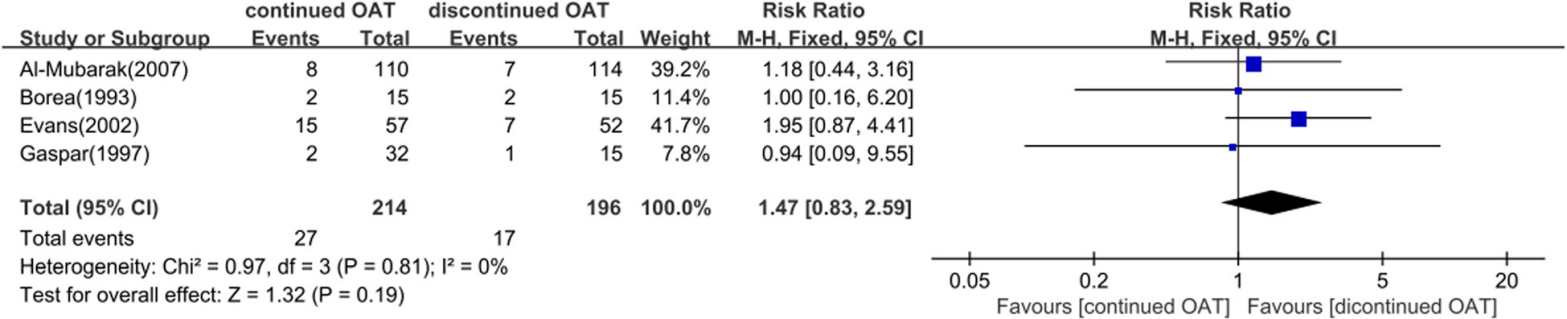
Forest plot of the difference of postoperative bleeding between OAT continued or discontinued group 7 days after the surgery

Four studies [4, 16, 21, 23], with 214 subjects in the treated group and 196 in the control group were included in the 7 day subgroup. The bleeding occurred in 27 of 214 (12.6 %) patients in the treated group and in 17 of 196 (8.7 %) in the control group. A fixed-effects model was applied and there was no significant difference between the two groups (P = 0.19). The risk ratio was 1.47 and 95 % CI (0.83, 2.59) (Fig. 5).

## Discussion

Numerous studies have reported that OAT reduces the risks of thromboembolism significantly [24, 25]. Other studies have documented that serious embolic complications, including death, are three times more likely to occur in patients whose anticoagulant therapy was interrupted, compared to patients with bleeding complications associated with anticoagulation [26]. For oral surgery procedures, no case of lethal postoperative bleeding in patients who continued OAT has been reported [16, 26], whereas several fatal thromboembolic events after stopping OAT for dental extractions have occurred [8, 27]. Nevertheless, there is still a lack of consensus regarding preoperative alteration of the anticoagulant regimen to prepare OAT patients for dental extractions.

### Summary of results

The results of our meta-analysis revealed that patients continuing oral anticoagulant therapy do not have an increased risk of bleeding after dental extraction compared to patients who discontinue oral anticoagulant therapy. However, the follow-up dates were not the same between these studies. One of the studies [22] only reported the first day bleeding outcome, and one study [7] followed-up for 5 days with a detailed record of everyday situations. The other four studies [4, 16, 21, 23] followed up for 7 days after the dental extraction, but three studies only mentioned the total number of bleeding events and the remaining one recorded everyday situations. Subgroup analyses were performed since three of the included studies [7, 21, 22] recorded the bleeding outcome 1 day after surgery, and four included studies [4, 16, 21, 23] collected measurements for 7 days after the surgery. The results were consistent with the previous results. There was no significant difference in bleeding risk between the OAT continued group and the OAT discontinued group 1 and 7 days after the surgery. Of all the studies, none of these patients suffered serious bleeding, and the bleeding was easily controlled by patients at home or controlled with local measures during their visit to the hospital. No thromboembolic event was reported in these studies, whether OAT was continued or not. However, this outcome could be a result of the short follow-up period of these studies (from 1 to 7 days), which made it difficult assess the thromboembolism risk in patients who discontinued OAT.

All of the 6 included studies came to the conclusion that OAT patients who do not discontinue the anticoagulant medication do not have a significantly higher risk of postoperative bleeding than OAT patients who stop the therapy. Some of the studies mentioned the importance of international normalized ratio (INR) and hemostatic procedures. INR has been used as a recommendation for monitoring patients’ oral anticoagulant therapy. For stroke prevention in atrial fibrillation patients, oral anticoagulation therapy that is dose adjusted to maintain an INR range of 2.0 to 3.0 is associated with a 64 % reduction in the risk of stroke compared to placebo [28]. And in patients suffering an acute venous thromboembolism (either deep vein thrombosis or pulmonary embolism), adjusted-dose OAT use significantly reduces the risk of recurrence of thrombotic events with a target INR range of 2.0–3.0. [29]. According to meta-analyses of atrial fibrillation or mixed populations assessing INR control and associated events [30], more than half of all thromboembolic events occurred when patients have an INR <2.0. Besides, the INR has a safe range before surgical procedures. This “safe range” is controversial because some experts recommended an INR of ≤3 [17, 31], whereas others suggest an INR of ≤4 [32, 33] as safe for dental extractions. An INR above 5 has been shown to be an unacceptable risk for postoperative bleeding [34]. However, none of the included patients had an INR of more than 5. Most of the patients had an INR no higher than 4, as shown in Table 3. Next to monitoring the INR, it is recommended to take a special care of patients with renal dysfunction. Given the renal excretion of drugs, renal dysfunction may result in a higher incidence of bleeding associated with oral anticoagulation [35, 36]. A variety of local hemostatic measures are used in oral surgeries, including sutures, local compression, adjuvants (such as fibrin and histoacryl glue), local antifibrinolytic solutions, collagen fleeces, acrylic splints, gelatin sponges and so on [37, 38]. Placing hemostatic, physiological or tranexamic acid irrigation and mouthwash were used in our included studies. However, one of the included studies [4] showed that suturing played no significant role in bleeding status. In their study, patients with sutures showed a higher incidence of bleeding than patients without sutures. This outcome might be caused by the mode of suturing that further traumatizes the soft tissue. Four of the included studies [7, 16, 21, 23] used local hemostatic measures, except for local compression and sutures. The effectiveness of these measures was worth studying.

### Limitations

There were some limitations in the included studies. Only one of the six included studies was judged to have a low risk of bias, and the absence of randomization may predispose the analysis to a risk of selection bias and possible confounding effects [39]. Some studies were carried out with limited patient numbers [21, 23] and, as with all meta-analyses, there may be studies published in other languages or unpublished studies that we were not able to access. Additionally, the INR differed between different studies which influenced the bleeding outcomes. Patients with a low INR were less likely to suffer bleeding than patients with a higher INR. Other factors, such as gingival health, numbers and the complexity of the tooth extractions, the surgical skills, anesthesia type, material and suture technique, local hemostatic measures, and the use of anticoagulants differed from one study to another or were not reported. This heterogeneity limited the scientific evidence that could be obtained for this review.

## Conclusion

Under current studies and evidence, the results of our meta-analysis revealed that patients continuing oral anticoagulant therapy do not increase the risk of bleeding after dental extractions compared to patients who discontinue oral anticoagulant therapy. However, with the limited number of included studies and the risk of bias, well-designed RCTs should be included with a larger sample size as well as specific inclusion and exclusion criteria. Moreover, it would be better if the bleeding outcomes had a reporting standard. This standardization would enhance the comparability of studies based on identical outcome measurements, which would help to establish guidelines for dentists who treat these patients with complex medical needs.

## Abbreviations

NOACs: New oral anticoagulants
OAT: Oral anticoagulant therapy
